# Annotations

**Published:** 1890-07-26

**Authors:** 


### Annotations.

**"Ye Odde Pye."**

The "Chelmsford Odd Volumes" are a kind of antiquarian society, the members of which, among other delights, indulge in long summer rambles to various places of local interest, and generally finish off their day with a modest hour of all-round feasting. At the close of their last expedition of this kind they were entertained in the castle grounds at Pleshey; and the *pièce de viande* was a pie of such unusual character that its composition may well be made known and considered by all who are interested in the development of high gastronomic art. This right Royal "pye" was prepared by Miss Eva Christy, and is stated to have contained, among other things, "field voles, hedgehogs, frogs, rabbits, fowls' eggs, beef, bacon, thrushes, blackbirds, greenfinches, sparrows, and seven fine specimens of the common barn rat, grain fed." The ingredients were kept a profound secret until the feast began, and even then were only revealed to the eye of the learned through the medium of a Latin menu. The unphilosophical stomach performs an unconscious shiver as it thinks of the hedgehogs, the frogs, the field voles, and the seven fine rats, grain fed; and possibly even the philosophic *fourchette* took care to pierce rather the loin of a rabbit or the safe fibres of the beef steak than the ham of a rat, or the pale shoulders of a cold-blooded frog. Nevertheless, it seems clear that the pie was eaten, for one enthusiastic visitor declares that he can "aver with truth" that the rats and hedgehogs were most appetizing, and that we have in these humble animals delicacies of the first order." The funny aspect of the case is that which will strike most readers; but there is no reason why we should not learn a lesson of a useful kind as well. There is probably not a farm labourer in England who does not know that frogs are eaten in France, that gipsies find hedgehogs most delicious picking, and that many a besieged town has held out till succour arrived on a diet composed largely of rats. The suggestion is that poor people might add both variety and piquancy to their fare by an occasional rat stew or frog patty. Rats and rabbits, as any student of elementary zoology can tell us, belong to the same order of creatures; and any rustic unbeliever may convince himself of this fact as he listens to an industrious rat gnawing through the lid or side of a corn bin. Both these creatures are rodents, and the flesh of the rat is as good as that of the rabbit, it may even be better when the former is "grain fed." It might be desirable, in order to convert the poor to sound views, for the better classes to take up the question in a practical way by having rat pie and other rat dishes at their tables from time to time.

**Lepers and Penny Whistles.**

"The doctor has removed all restrictions as to mouth-organs, and the patients love anything which will make a noise, from a penny whistle upwards." Most readers will remember the outcry which was made about the lepers of Robben Island a few months ago. The island is situated off the South African Coast, and it is used to some extent as a sanatorium for lepers. However much or little truth there may have been in the distressing reports of neglect and hardship formerly endured by the unfortunate sufferers who had been sent to the island, it is quite certain that now, at any rate, very active measures are being taken by influential persons for their comfort and well-being. A private letter addressed to the Rev. W. T. McCormick, vicar of St. Matthew's, Brighton, has been forwarded to us, with the object of interesting the British public on their behalf. Lepers are still lepers, in spite of all that can be done for them; and the truest humanity consists in using every possible means for the alleviation of their condition, and for making life as bright and cheery for them as the possibilities will allow. They are fond of music; and at this nobody will be surprised who has experienced its soothing and inspiriting power in times of distress and calamity. Visitors from the mainland of South Africa, who include among their number Lady Loch, Lady Gordon Sprigg, and several other married and unmarried ladies, have arranged to periodically visit the island and to personally carry to each individual sufferer any gifts with which they may be entrusted by benevolent persons in this country or in Africa. Those kind ladies have struck out a new line of operations. They are not going merely to pay a patronising visit to their protégés, but they are going to talk, and still better, to sing to them. The "singing" is an excellent new departure. The moment a kind-hearted woman has begun to sing for another, she has brought herself miles nearer to that other's sympathy and affection. The music is a bridge which unites the two in a common humanity and pleasure. But if we, the writer and the reader, try to imagine ourselves paying a round of visits to a dozen or a score lepers and other sick persons on a remote island, we shall feel at once how doubly welcome our visit would be, if we could not only contribute a lively song or two, but a few ounces of tobacco for the men, or a pound or two of good tea for the women. As a matter of fact, this is what the Rev. Charles Simeon would have called the "application" of, or little sermon. The lady and gentlemen visitors are anxious to go full-handed to their poor friends; and they cannot do this if they always have to put their hands into their own pockets. Generous persons in this country will feel that it is a good thing that those ladies and gentlemen are on the spot, and are willing to do a little wholesome gossiping and singing with the lepers, and to act as almoners as well. We, for our part, will do the begging, and in order to do it successfully, we give a list of a few things which the ladies and the doctor tell us will be most acceptable to the sufferers. First of all a magic lantern, with a great variety of slides, is asked for. Next in order are tobacco, sweetmeats and sweet biscuits, tea, coffee and sugar, pictures, books and wall texts, cardigan waistcoats, muffs, wooden pipes, ribbons, trinkets, and toys (for there are about a dozen children there), games of all sorts, hammocks, and last, but not least, old pianos. When it is added that this charitable movement is entirely unsectarian, and that there are on the general committee, among others, Archdeacon Lightfoot, of the English Church; the Rev. W. Forbes, Congregational minister; the Rev. J. Turnbull, of the Dutch Church; T. E. Fuller, M.P.; and Surgeon Truman, of the Military Hospital, all intending givers, may be assured of the impartial distribution of their gifts. Communications may be made to Miss Clara Boyes, secretary to the Robben Island Relief Fund, Cape Town, South Africa.

**A Medical School for Wales.**

Scotland has seven or eight medical schools, and Ireland has almost as many. Wales cannot boast of so much as one. It is not that Wales is so healthy as not to need doctors, or that Welshmen are so much behind the times as not to study medicine scientifically. The circumstance seems to be rather of that accidental kind which admits of no one explanation in particular. But a school of medicine Wales is determined to have now, as is proved by the meeting over which Lord Aberdare presided at the rooms of the Royal Medical and Chirurgical Society on Wednesday last. The medical school is, of course, to be associated with the University College of South Wales and Monmouthshire. The idea, no doubt, is to have an university of all the faculties. A medical faculty is imperatively demanded for Wales, and it will certainly be created. It may be hoped, however, that those who take the subject in hand will consider themselves free to found a school on rational and practical lines, and will not be bound by the traditions and ways of other institutions. A school of medicine, like any other school, is first and chiefly a place of instruction. Surely, then, the first thing to do is to get together a number of persons who, above all things, can teach. The school should have a really responsible head, capable both of directing studies and of inspiring alike the teachers and the taught. In England and in Scotland, too, there are all sorts of dull men presiding over classes of students, and making medical study the least intellectual, the least practical, and the least tolerable of all the studies in which the youth of Britain are engaged. Why is this? It is because hospital authorities are so insane as to appoint men on the strength of their degrees alone, without thinking at all whether they can teach or not. This is much the same as appointing a postman merely because he has black hair, and without taking care to notice if he be also endowed with legs. Unless we are to suppose that hospital physicians, surgeons, and managers have so little confidence in the medical art that they do not care at all how it is taught, it is difficult, nay almost impossible, to understand their mental attitude on the subject. If a surgeon be chosen because he is a skilled operator, and a physician because he is a successful prescriber, it is surely but the application of the most elementary common sense to choose a teacher because he can teach.

